# (*RSS*)-[*N*-Hydroxyethyloxy]-hexafluoroVal–MeLeu–Ala *tert*-butyl ester

**DOI:** 10.1107/S1600536809042974

**Published:** 2009-10-28

**Authors:** Marcel K. Eberle, Helen Stoeckli-Evans, Reinhart Keese

**Affiliations:** a Department of Chemistry and Biochemistry, University Bern, Freiestrasse 3, CH-3012 Bern, Switzerland; b Institute of Physics, University of Neuchâtel, rue Emile-Argand 11, CH-2009 Neuchâtel, Switzerland

## Abstract

The title compound [systematic name: (2*S*,5*S*,8*R*)-*tert*-butyl 8-(1,1,1,3,3,3-hexafluoropropan-2-yl)-12-hydroxy-5-isobutyl-2,6-dimethyl-4,7-dioxo-10-oxa-3,6,9-triazadodecanoate], C_21_H_36_F_6_N_3_O_6_, is a tripeptide crystallizing in the chiral ortho­rhom­bic spacegroup *P*2_1_2_1_2_1_. The absolute configuration (*R*) of the chiral center in the hexa­fluoro­valine unit is based on the known stereochemistry of MeLeu and Ala (*SS*). The *N*-hydroxy­ethyl­oxy substituent of hexa­fluoro­valine is positionally disordered [occupancy ratio 0.543 (9):0.457 (9)]. In the solid state structure there are N—H⋯F and N—H⋯O intra­molecular hydrogen bonds supporting the coiled structure of this tripeptide with the three hydro­phobic substituents on the outside.

## Related literature

For biomolecules with fluoro substituents, see: Kirsch (2004 ▶); Mikol *et al.* (1997 ▶); Eberle *et al.* (1998 ▶); Zhang *et al.* (1998 ▶); Eberle & Keese (2009 ▶). For the tripeptide Val-MeLeu-Ala in cyclo­sporine, an undeca­peptide, and the fact that it can be extracted and reintroduced in the remaining octa­peptide, see: Eberle *et al.* (1994 ▶).
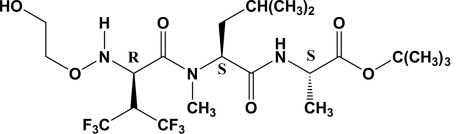


## Experimental

### 

#### Crystal data

C_21_H_35_F_6_N_3_O_6_
*M* *_r_* = 539.52
Orthorhombic, 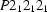

*a* = 11.1608 (7) Å
*b* = 11.2088 (7) Å
*c* = 21.2562 (15) Å
*V* = 2659.1 (3) Å^3^
*Z* = 4
Mo *K*α radiation
μ = 0.12 mm^−1^
*T* = 153 K
0.50 × 0.50 × 0.40 mm

#### Data collection

Stoe IPDS diffractometer
Absorption correction: none
18889 measured reflections
2917 independent reflections
2554 reflections with *I* > 2σ(*I*)
*R*_int_ = 0.063

#### Refinement

*R*[*F* ^2^ > 2σ(*F* ^2^)] = 0.077
*wR*(*F* ^2^) = 0.201
*S* = 1.14
2917 reflections
358 parameters
8 restraints
H atoms treated by a mixture of independent and constrained refinement
Δρ_max_ = 0.54 e Å^−3^
Δρ_min_ = −0.37 e Å^−3^

### 

Data collection: *EXPOSE* in *IPDS-I* (Stoe & Cie, 2000 ▶); cell refinement: *CELL* in *IPDS-I*; data reduction: *INTEGRATE* in *IPDS-I*; program(s) used to solve structure: *SHELXS97* (Sheldrick, 2008 ▶); program(s) used to refine structure: *SHELXL97* (Sheldrick, 2008 ▶); molecular graphics: *PLATON* (Spek, 2009 ▶); software used to prepare material for publication: *SHELXL97*.

## Supplementary Material

Crystal structure: contains datablocks I, global. DOI: 10.1107/S1600536809042974/im2143sup1.cif

Structure factors: contains datablocks I. DOI: 10.1107/S1600536809042974/im2143Isup2.hkl

Additional supplementary materials:  crystallographic information; 3D view; checkCIF report

## Figures and Tables

**Table 1:** Hydrogen-bond geometry (Å, °)

*D*—H⋯*A*	*D*—H	H⋯*A*	*D*⋯*A*	*D*—H⋯*A*
N3*A*—H3*A*1⋯F5	0.88	1.94	2.60 (3)	130
N1—H1*N*⋯O2	0.88 (5)	2.37 (7)	2.720 (9)	104 (5)
N1—H1*N*⋯O5*A*	0.88 (5)	2.38 (6)	3.245 (17)	169 (6)

**Fig. 1.**
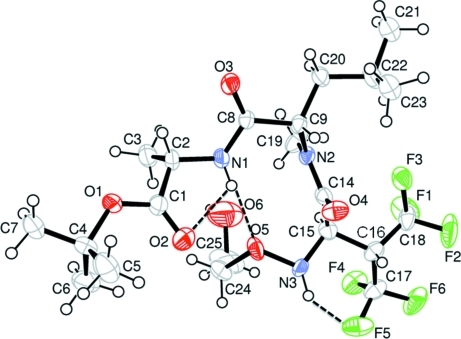
The molecular structure of the title compound with displacement ellipsoids drawn at the 50% probability level. The intramolecular N—H···F and N—H···O hydrogen bonds are shown as dashed lines [The B fractions of the disordered –NH—O—CH2—CH2OH side chain and a methyl group of the isopropyl substituent have been omitted for clarity].

**Fig. 2.**
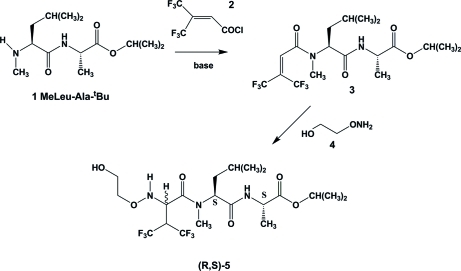
The preparation of the title compound.

## supplementary crystallographic information

### Comment

Biomolecules with fluoro substituents differ in their properties compared to their congeners by a variety of properties. For example, they tend to be more lipophilic, exhibit a higher stability and may excert specific medicinal effects (Kirsch, 2004; Mikol *et al.*, 1997). As part of our interest in fluorinated biomolecules we have prepared (*R*)- and (*S*)-4,4,4,4',4',4'-hexafluorovaline (Eberle *et al.*, 1998; Eberle & Keese, 2009) and (*S*)-5,5,5,5',5,'5'-hexafluoroleucine (Zhang *et al.*, 1998). Our interest was further stimulated when we realised that cyclosporine, an undecapeptide and an important immunosuppressant, contains (*S*)-valine as one of the 11 aminoacids (Mikol *et al.*, 1997). Furthermore, earlier work had shown that the tripeptide Val-MeLeu-Ala can be chemically cut out of the undecapeptide and reintroduced eventually into the remaining octapeptide (Eberle *et al.*, 1994).

In order to replace Val in the tripeptide Val-MeLeu-Ala by hexafluoro-valine the depsipeptide, (*S*)-1*N*-methyl-leucine-(*S*)-alanine-*O*-*tert*-butylester-1*N-*(3,3-bistrifluoromethyl-acryloylamide, was prepared and subsequently the title tripeptide containing an *N*-functionalized 4,4,4,4',4',4'-hexafluorovaline. The depsipeptide was obtained from (*S*)-*N*-methylleucine-(*S*)-alanine-*O*-*tert*-butylester by acylation with 4,4,4,4',4',4'-hexafluoro acrylic acid. Subsequent addition of 2-hydroxyethyloxyamine in α-position of the hexafluoroacrylic moiety in the depsipeptide gave two diastereomers of the title tripeptide. One of these solidified and gave crystals suitable for an X-ray structure analysis, and we describe its crystal structure herein.

Based on the known absolute configuration of MeLeu and Ala, (*SS*), used in the synthesis, the hexafluoro-valine moiety in the title compound has (*R*)-configuration (Fig. 1). The geometrical parameters are available in the archived CIF. The –NH—O—CH_2_—CH_2_OH side chain of the hexafluoro Valine is disordered over two positions [occupancies *A*:*B* = 0.6:0.4]. One methyl group (C23) of the isopropyl substituent in the central amino acid MeLeu is also disordered over two positions [occupancies *A*:*B* = 0.5:0.5]. The bond angle in the hexafluoroisopropyl group (C17—C16—C18) in hexafluoro valine is 109.3 (6)°, whereas the bond angles in the isopropyl group of MeLeu within the two orientations (C21—C22—C23A and C21—C22—C23B) are 107.5 (9)° and 111.8 (10)°, respectively. The bond angle C9—C20—C22 of the leucine side chain is 114.7 (4)°. The bond angles at the acylated N-atoms, N1 and N2, are close to 120°, the value expected for tricoordinate *sp*^2^ hybridized N-atoms. The torsional angles around the two amide groups [2.2 (10)° for C2—N1—C8—O3, 178.7 (6) ° for C2—N1—C8—C9, and -1.8 (9) ° for C9—N2—C14—O4, 173.6 (5) ° for C9—N2—C14—C15] are within the normal range and provide the evidence for almost planar arrangements (Fig. 1).

In the crystal structure there is a three-centered hydrogen bond located between N3—H3N···F5, whereas a bifurcated hydrogen bond O2—H1N—O5 leads to interactions between the N1—H and atom O5, the N-bonded O-atom of the hydroxyethyloxy side chain, and the carbonyl group of the BOC protecting group (Table 1 and Fig. 1). These intramolecular hydrogen bonds support the coiled structure of this tripeptide with the three hydrophobic substituents on the outside.

### Experimental

The synthesis of the title compound is summerized in Fig. 2, and full details will be published elsewhere (Eberle & Keese, 2009). In order to replace Val by hexafluoro-valine in the tripeptide Val-MeLeu-Ala, the depsipeptide (3), and subsequently the tripeptide (*RS*)-(5) containing an *N*-functionalized 4,4,4,4',4',4'-hexafluorovaline, were prepared. Compound (3) was obtained from (*S*)-*N*-methylleucine-(*S*)-alanine-*O*-*tert*-butylester (1) by acylation with 4,4,4,4',4',4'-hexafluoro acrylic acid (2). The subsequent addition of 2-hydroxyethyloxyamine (4) to the α-position of the hexafluoroacrylic moiety in (3) gave two diastereomers of the title tripeptide (*RS*)-(5). One of these solidified and crystals, suitable for X-ray analysis, were obtained as colourless rods from ether-hexane (1:5) on slow evaporation at room temperature.

### Refinement

In the final cycles of refinement, in the absence of significant anomalous scattering effects, the 2234 Friedel pairs were merged and Δf " set to zero. The coordinates correspond to the absolute structure of the molecule in the crystal assigned with reference to the known stereospecific centers in the molecule. The –NH—O—CH_2_—CH_2_OH side chain is disordered over two positions (occupancies A:B = 0.6:0.4). One methyl group (C23) of the isopropyl substituent is also disordered over two positions (occupancies A:B = 0.5:0.5). During refinement a certain number of restraints were applied: ADP's of the pairs of atoms C23A & C21, C23B & C21, C24B & C24A, C25B & C25A were made equal; bonds C22—C23A, C22—C23B, C24B—C25B, C24A—C25A were refined with distance restraints of 1.54 (2) Å and bonds C25A—O6A, C25B—O6B with distant restraints of 1.44 (2) Å. The N—H hydrogen atoms could be located in difference Fourier syntheses. N1—H1N was freely refined (N—H = 0.88 (4) Å), while N3a—H3a was included in the calculated position and treated as riding: N—H = 0.88 Å with *U*_iso_(H) = 1.2*U*_eq_(N-atom). The O– and C-bound H-atoms were included in calculated positions and treated as riding atoms: O—H = 0.84 Å, C—H = 0.98 - 1.00 Å with *U*_iso_(H) = 1.5*U*_eq_(parent O-atom) and = 1.2*U*_eq_(parent C-atom).

### Crystal data

**Table:**

C_21_H_35_F_6_N_3_O_6_	*F*(000) = 1136
*M**_r_* = 539.52	*D*_x_ = 1.348 Mg m^−^^3^
Orthorhombic, *P*2_1_2_1_2_1_	Mo *K*α radiation, λ = 0.71073 Å
Hall symbol: P 2ac 2ab	Cell parameters from 8000 reflections
*a* = 11.1608 (7) Å	θ = 2.1–25.9°
*b* = 11.2088 (7) Å	µ = 0.12 mm^−^^1^
*c* = 21.2562 (15) Å	*T* = 153 K
*V* = 2659.1 (3) Å^3^	Rod, colourless
*Z* = 4	0.50 × 0.50 × 0.40 mm

### Data collection

**Table:**

Stoe IPDS diffractometer	2554 reflections with *I* > 2σ(*I*)
Radiation source: fine-focus sealed tube	*R*_int_ = 0.063
graphite	θ_max_ = 25.9°, θ_min_ = 2.1°
phi oscillation scans	*h* = −13→13
18889 measured reflections	*k* = −13→13
2917 independent reflections	*l* = −26→25

### Refinement

**Table:**

Refinement on *F*^2^	Secondary atom site location: difference Fourier map
Least-squares matrix: full	Hydrogen site location: inferred from neighbouring sites
*R*[*F*^2^ > 2σ(*F*^2^)] = 0.077	H atoms treated by a mixture of independent and constrained refinement
*wR*(*F*^2^) = 0.201	*w* = 1/[σ^2^(*F*_o_^2^) + (0.0533*P*)^2^ + 7.6569*P*] where *P* = (*F*_o_^2^ + 2*F*_c_^2^)/3
*S* = 1.14	(Δ/σ)_max_ < 0.001
2917 reflections	Δρ_max_ = 0.54 e Å^−^^3^
358 parameters	Δρ_min_ = −0.37 e Å^−^^3^
8 restraints	Extinction correction: *SHELXL*, Fc^*^=kFc[1+0.001xFc^2^λ^3^/sin(2θ)]^-1/4^
Primary atom site location: structure-invariant direct methods	Extinction coefficient: 0.010 (2)

### Special details

**Table:**

Geometry. Bond distances, angles etc. have been calculated using the rounded fractional coordinates. All su's are estimated from the variances of the (full) variance-covariance matrix. The cell esds are taken into account in the estimation of distances, angles and torsion angles
Refinement. In the final cycles of refinement, in the absence of significant anomalous scattering effects, the 2234 Friedel pairs were merged and Δf " set to zero. The –NH—O—CH_2_CH_2_OH side chain is disordered over two positions (A & B: occupancies 0.6 / 0.4). One methyl group (C23) of the isopropyl substituent is disordered over two positions (A & B: occupancies 0.5 / 1/2). The coordinates correspond to the absolute structure of the molecule in the crystal, assigned with reference to the known stereospecific centers in the molecule. During refinement a certain number of restraints were applied, for example, EADP N3A N3B; EADP C23A C21 EADP C23B C21; EADP C25A C25B C24B C24A; DFIX 1.54 .02 C22 C23A C22 C23B C24B C25B C24A C25A; DFIX 1.44 .02 C25A O6A C25B O6B; DFIX 0.88 .02 N1 H1N; DFIX -2.2 .02 H9 H23A; DFIX -3.0 .02 C23A O6B.

### Fractional atomic coordinates and isotropic or equivalent isotropic displacement parameters (Å^2^)

**Table:**

	*x*	*y*	*z*	*U*_iso_*/*U*_eq_	Occ. (<1)
F1	0.8576 (6)	0.5625 (5)	0.7742 (2)	0.0700 (19)	
F2	1.0348 (6)	0.6382 (5)	0.7580 (2)	0.083 (2)	
F3	0.9114 (5)	0.7225 (4)	0.82195 (18)	0.0505 (14)	
F4	0.9183 (4)	0.3504 (4)	0.8362 (2)	0.0513 (16)	
F5	1.0984 (5)	0.3567 (4)	0.8715 (3)	0.0623 (18)	
F6	1.0617 (5)	0.4058 (4)	0.7766 (2)	0.0670 (19)	
O1	0.9061 (4)	0.6526 (4)	1.24644 (19)	0.0343 (12)	
O2	0.9956 (5)	0.6041 (6)	1.1539 (3)	0.0573 (19)	
O3	0.7713 (5)	0.9284 (4)	1.0905 (2)	0.0357 (14)	
O4	1.0307 (4)	0.7163 (4)	0.9528 (2)	0.0403 (17)	
O5A	0.9190 (14)	0.4938 (14)	1.0180 (7)	0.042 (4)	0.543 (9)
O6A	0.6768 (14)	0.4094 (14)	1.0107 (12)	0.127 (8)	0.543 (9)
N1	0.8327 (6)	0.7356 (5)	1.0887 (3)	0.0387 (19)	
N2	0.8262 (5)	0.7377 (5)	0.9574 (2)	0.0270 (16)	
N3A	0.982 (2)	0.473 (3)	0.9578 (16)	0.031 (6)	0.543 (9)
C1	0.9169 (7)	0.6529 (6)	1.1836 (3)	0.0350 (19)	
C2	0.8107 (6)	0.7148 (6)	1.1547 (3)	0.0337 (19)	
C3	0.6980 (7)	0.6393 (8)	1.1630 (3)	0.045 (3)	
C4	0.9872 (7)	0.5833 (7)	1.2875 (3)	0.039 (2)	
C5	1.1153 (8)	0.6268 (9)	1.2817 (4)	0.061 (3)	
C6	0.9761 (9)	0.4527 (7)	1.2725 (4)	0.060 (3)	
C7	0.9347 (8)	0.6062 (8)	1.3525 (3)	0.050 (3)	
C8	0.8089 (5)	0.8426 (6)	1.0615 (3)	0.0260 (17)	
C9	0.8399 (6)	0.8523 (5)	0.9904 (3)	0.0293 (17)	
C14	0.9274 (7)	0.6796 (6)	0.9424 (3)	0.0337 (19)	
C15	0.9200 (7)	0.5526 (6)	0.9140 (3)	0.0330 (19)	
C16	1.0017 (7)	0.5429 (6)	0.8559 (3)	0.036 (2)	
C17	1.0167 (7)	0.4139 (7)	0.8347 (4)	0.041 (2)	
C18	0.9518 (8)	0.6150 (7)	0.8028 (3)	0.044 (2)	
C19	0.7068 (6)	0.6926 (7)	0.9431 (4)	0.044 (2)	
C20	0.7694 (7)	0.9544 (6)	0.9583 (3)	0.0357 (19)	
C21	0.7368 (9)	1.0861 (8)	0.8671 (4)	0.056 (3)	
C22	0.8160 (9)	0.9903 (7)	0.8948 (4)	0.059 (3)	
C23A	0.9457 (15)	1.0506 (16)	0.9098 (8)	0.056 (3)	0.500
C23B	0.9385 (14)	1.0120 (17)	0.8825 (8)	0.056 (3)	0.500
C24A	0.8850 (19)	0.3851 (14)	1.0521 (10)	0.080 (4)	0.543 (9)
C25A	0.7784 (17)	0.3265 (15)	1.0138 (12)	0.080 (4)	0.543 (9)
N3B	0.947 (3)	0.465 (4)	0.966 (2)	0.031 (6)	0.457 (9)
O5B	0.8575 (18)	0.4829 (19)	1.0142 (11)	0.059 (6)	0.457 (9)
O6B	0.9684 (16)	0.2308 (12)	1.0006 (7)	0.073 (6)	0.457 (9)
C24B	0.803 (2)	0.3747 (19)	1.0197 (15)	0.080 (4)	0.457 (9)
C25B	0.882 (2)	0.2763 (17)	1.0439 (11)	0.080 (4)	0.457 (9)
H3A1	1.04260	0.42520	0.95020	0.0380*	0.543 (9)
H3C	0.67350	0.64060	1.20730	0.0680*	
H5A	1.11730	0.71370	1.28630	0.0920*	
H1N	0.860 (7)	0.667 (4)	1.075 (3)	0.0470*	
H2	0.79870	0.79330	1.17620	0.0400*	
H6A1	0.61320	0.37270	1.01920	0.1510*	0.543 (9)
H3A	0.63350	0.67190	1.13690	0.0680*	
H3B	0.71470	0.55700	1.15020	0.0680*	
H7A	0.85130	0.57900	1.35360	0.0760*	
H7B	0.93780	0.69180	1.36170	0.0760*	
H7C	0.98130	0.56260	1.38410	0.0760*	
H9	0.92660	0.87400	0.98770	0.0350*	
H15	0.83580	0.52690	0.90530	0.0400*	
H16	1.08230	0.57540	0.86710	0.0430*	
H19A	0.69300	0.69650	0.89760	0.0660*	
H19B	0.64680	0.74140	0.96480	0.0660*	
H19C	0.70030	0.60970	0.95730	0.0660*	
H20A	0.68480	0.92950	0.95380	0.0430*	
H20B	0.77110	1.02510	0.98630	0.0430*	
H21A	0.65580	1.05440	0.86110	0.0840*	
H21B	0.73380	1.15450	0.89580	0.0840*	
H21C	0.76950	1.11160	0.82650	0.0840*	
H22A	0.82370	0.92020	0.86600	0.0710*	0.500
H22B	0.80410	0.91870	0.86740	0.0710*	0.500
H23A	1.00580	0.98780	0.91550	0.0840*	0.500
H23B	0.94010	1.09830	0.94840	0.0840*	0.500
H23C	0.96930	1.10210	0.87470	0.0840*	0.500
H23D	0.96630	1.07870	0.90850	0.0840*	0.500
H23E	0.94910	1.03180	0.83800	0.0840*	0.500
H23F	0.98520	0.94050	0.89260	0.0840*	0.500
H24A	0.85890	0.40490	1.09540	0.0950*	0.543 (9)
H24B	0.95370	0.32950	1.05460	0.0950*	0.543 (9)
H25A	0.80560	0.30680	0.97070	0.0950*	0.543 (9)
H25B	0.75300	0.25160	1.03460	0.0950*	0.543 (9)
H5B	1.14720	0.60460	1.24040	0.0920*	
H5C	1.16430	0.59010	1.31480	0.0920*	
H6A	1.00380	0.43820	1.22940	0.0900*	
H6B	0.89210	0.42830	1.27640	0.0900*	
H6C	1.02510	0.40650	1.30200	0.0900*	
H3B1	1.00600	0.41330	0.96670	0.0380*	0.457 (9)
H6B1	0.97770	0.27970	0.97100	0.0880*	0.457 (9)
H24C	0.77180	0.35110	0.97790	0.0950*	0.457 (9)
H24D	0.73370	0.38290	1.04840	0.0950*	0.457 (9)
H25C	0.92560	0.30610	1.08140	0.0950*	0.457 (9)
H25D	0.83080	0.20940	1.05780	0.0950*	0.457 (9)

### Atomic displacement parameters (Å^2^)

**Table:**

	*U*^11^	*U*^22^	*U*^33^	*U*^12^	*U*^13^	*U*^23^
F1	0.098 (4)	0.064 (3)	0.048 (3)	−0.004 (3)	−0.034 (3)	−0.008 (2)
F2	0.138 (6)	0.066 (3)	0.044 (3)	−0.011 (4)	0.050 (3)	−0.005 (2)
F3	0.083 (3)	0.040 (2)	0.0286 (19)	0.005 (3)	0.007 (2)	−0.0019 (17)
F4	0.054 (3)	0.035 (2)	0.065 (3)	−0.011 (2)	0.003 (2)	−0.018 (2)
F5	0.057 (3)	0.039 (2)	0.091 (4)	0.011 (2)	−0.003 (3)	−0.017 (3)
F6	0.085 (4)	0.056 (3)	0.060 (3)	−0.002 (3)	0.033 (3)	−0.027 (3)
O1	0.039 (2)	0.036 (2)	0.028 (2)	0.008 (2)	−0.005 (2)	0.0023 (19)
O2	0.053 (3)	0.075 (4)	0.044 (3)	0.024 (3)	0.009 (3)	0.007 (3)
O3	0.053 (3)	0.029 (2)	0.025 (2)	−0.001 (2)	0.003 (2)	−0.0058 (19)
O4	0.038 (3)	0.035 (3)	0.048 (3)	0.003 (2)	−0.010 (2)	−0.018 (2)
O5A	0.074 (10)	0.029 (5)	0.022 (5)	0.000 (8)	0.006 (7)	0.002 (4)
O6A	0.078 (10)	0.082 (10)	0.22 (2)	0.012 (9)	0.051 (13)	0.056 (13)
N1	0.062 (4)	0.028 (3)	0.026 (3)	0.008 (3)	0.011 (3)	0.003 (2)
N2	0.034 (3)	0.026 (3)	0.021 (2)	−0.003 (2)	0.005 (2)	−0.003 (2)
N3A	0.030 (13)	0.035 (6)	0.029 (9)	0.004 (11)	0.016 (9)	0.004 (5)
C1	0.040 (4)	0.035 (3)	0.030 (3)	0.002 (3)	0.001 (3)	0.001 (3)
C2	0.042 (4)	0.037 (3)	0.022 (3)	0.003 (3)	0.001 (3)	0.003 (3)
C3	0.042 (4)	0.054 (5)	0.040 (4)	0.003 (4)	0.006 (3)	0.014 (4)
C4	0.036 (4)	0.040 (4)	0.040 (4)	0.011 (3)	−0.005 (3)	0.006 (3)
C5	0.054 (5)	0.073 (6)	0.057 (5)	−0.007 (5)	−0.014 (4)	0.018 (5)
C6	0.073 (6)	0.040 (4)	0.068 (6)	0.016 (4)	−0.002 (5)	0.007 (4)
C7	0.063 (5)	0.051 (5)	0.037 (4)	0.007 (4)	−0.005 (4)	0.009 (3)
C8	0.029 (3)	0.027 (3)	0.022 (3)	−0.003 (3)	0.001 (2)	−0.001 (2)
C9	0.038 (3)	0.025 (3)	0.025 (3)	−0.002 (3)	0.007 (3)	−0.003 (3)
C14	0.049 (4)	0.030 (3)	0.022 (3)	0.006 (3)	−0.003 (3)	0.001 (3)
C15	0.040 (4)	0.026 (3)	0.033 (3)	−0.003 (3)	0.002 (3)	−0.002 (3)
C16	0.039 (4)	0.037 (4)	0.032 (3)	0.001 (3)	0.004 (3)	−0.011 (3)
C17	0.039 (4)	0.034 (4)	0.051 (4)	−0.004 (3)	0.005 (3)	−0.014 (3)
C18	0.065 (5)	0.039 (4)	0.028 (3)	−0.009 (4)	0.012 (3)	−0.009 (3)
C19	0.039 (4)	0.042 (4)	0.050 (4)	−0.006 (3)	0.008 (4)	−0.018 (3)
C20	0.051 (4)	0.026 (3)	0.030 (3)	0.000 (3)	0.002 (3)	0.002 (3)
C21	0.074 (5)	0.051 (4)	0.044 (4)	0.007 (4)	0.003 (4)	0.013 (3)
C22	0.107 (8)	0.031 (4)	0.038 (4)	0.009 (5)	0.016 (5)	0.008 (3)
C23A	0.074 (5)	0.051 (4)	0.044 (4)	0.007 (4)	0.003 (4)	0.013 (3)
C23B	0.074 (5)	0.051 (4)	0.044 (4)	0.007 (4)	0.003 (4)	0.013 (3)
C24A	0.093 (8)	0.038 (5)	0.108 (8)	0.016 (6)	0.024 (7)	0.034 (7)
C25A	0.093 (8)	0.038 (5)	0.108 (8)	0.016 (6)	0.024 (7)	0.034 (7)
N3B	0.030 (13)	0.035 (6)	0.029 (9)	0.004 (11)	0.016 (9)	0.004 (5)
O5B	0.080 (14)	0.037 (9)	0.060 (10)	0.013 (11)	0.029 (12)	−0.002 (7)
O6B	0.102 (12)	0.043 (8)	0.074 (9)	0.028 (8)	0.018 (9)	0.019 (7)
C24B	0.093 (8)	0.038 (5)	0.108 (8)	0.016 (6)	0.024 (7)	0.034 (7)
C25B	0.093 (8)	0.038 (5)	0.108 (8)	0.016 (6)	0.024 (7)	0.034 (7)

### Geometric parameters (Å, °)

**Table:**

F1—C18	1.350 (10)	C24B—C25B	1.50 (3)
F2—C18	1.354 (10)	C2—H2	1.0000
F3—C18	1.349 (9)	C3—H3A	0.9800
F4—C17	1.309 (9)	C3—H3C	0.9800
F5—C17	1.362 (10)	C3—H3B	0.9800
F6—C17	1.336 (10)	C5—H5B	0.9800
O1—C1	1.341 (8)	C5—H5A	0.9800
O1—C4	1.478 (9)	C5—H5C	0.9800
O2—C1	1.212 (9)	C6—H6B	0.9800
O3—C8	1.217 (8)	C6—H6A	0.9800
O4—C14	1.244 (9)	C6—H6C	0.9800
O5A—N3A	1.48 (4)	C7—H7C	0.9800
O5A—C24A	1.47 (2)	C7—H7A	0.9800
O5B—C24B	1.36 (3)	C7—H7B	0.9800
O5B—N3B	1.45 (4)	C9—H9	1.0000
O6A—C25A	1.47 (2)	C15—H15	1.0000
O6B—C25B	1.43 (3)	C16—H16	1.0000
O6A—H6A1	0.8400	C19—H19B	0.9800
O6B—H6B1	0.8400	C19—H19A	0.9800
N1—C8	1.358 (9)	C19—H19C	0.9800
N1—C2	1.443 (9)	C20—H20B	0.9900
N2—C19	1.457 (9)	C20—H20A	0.9900
N2—C14	1.342 (9)	C21—H21A	0.9800
N2—C9	1.472 (8)	C21—H21B	0.9800
N3A—C15	1.46 (3)	C21—H21C	0.9800
N3B—C15	1.51 (4)	C22—H22A	1.0000
N1—H1N	0.88 (5)	C22—H22B	1.0000
N3A—H3A1	0.8800	C23A—H23C	0.9800
N3B—H3B1	0.8800	C23A—H23D	0.3900
C1—C2	1.505 (10)	C23A—H23B	0.9800
C2—C3	1.526 (11)	C23A—H23A	0.9800
C4—C6	1.503 (11)	C23A—H23F	1.3600
C4—C7	1.523 (10)	C23B—H23C	1.0800
C4—C5	1.516 (12)	C23B—H23A	1.0600
C8—C9	1.554 (9)	C23B—H23F	0.9800
C9—C20	1.547 (9)	C23B—H23D	0.9800
C14—C15	1.548 (9)	C23B—H23E	0.9800
C15—C16	1.539 (10)	C24A—H24A	0.9900
C16—C17	1.524 (10)	C24A—H24B	0.9900
C16—C18	1.496 (10)	C24B—H24C	0.9900
C20—C22	1.501 (11)	C24B—H24D	0.9900
C21—C22	1.510 (13)	C25A—H25A	0.9900
C22—C23B	1.413 (19)	C25A—H25B	0.9900
C22—C23A	1.63 (2)	C25B—H25C	0.9900
C23A—C23B	0.73 (2)	C25B—H25D	0.9900
C24A—C25A	1.58 (3)		
			
C1—O1—C4	122.3 (5)	C4—C7—H7B	109.00
N3A—O5A—C24A	114.8 (18)	N2—C9—H9	107.00
N3B—O5B—C24B	104 (3)	C8—C9—H9	107.00
C25A—O6A—H6A1	109.00	C20—C9—H9	107.00
C25B—O6B—H6B1	109.00	C14—C15—H15	113.00
C2—N1—C8	121.6 (6)	N3A—C15—H15	113.00
C9—N2—C19	119.8 (5)	C16—C15—H15	113.00
C9—N2—C14	116.7 (6)	N3B—C15—H15	98.00
C14—N2—C19	123.5 (6)	C15—C16—H16	108.00
O5A—N3A—C15	103.3 (18)	C18—C16—H16	109.00
O5B—N3B—C15	107 (3)	C17—C16—H16	109.00
C8—N1—H1N	135 (4)	N2—C19—H19C	109.00
C2—N1—H1N	104 (4)	N2—C19—H19B	109.00
C15—N3A—H3A1	128.00	H19B—C19—H19C	109.00
O5A—N3A—H3A1	128.00	H19A—C19—H19B	109.00
C15—N3B—H3B1	126.00	H19A—C19—H19C	110.00
O5B—N3B—H3B1	127.00	N2—C19—H19A	110.00
O1—C1—O2	125.6 (7)	C22—C20—H20B	109.00
O1—C1—C2	109.7 (6)	H20A—C20—H20B	108.00
O2—C1—C2	124.5 (6)	C9—C20—H20B	108.00
C1—C2—C3	110.3 (6)	C22—C20—H20A	109.00
N1—C2—C3	110.0 (5)	C9—C20—H20A	109.00
N1—C2—C1	109.7 (6)	C22—C21—H21A	109.00
C5—C4—C6	112.0 (7)	C22—C21—H21B	109.00
O1—C4—C7	102.2 (6)	H21B—C21—H21C	109.00
O1—C4—C6	109.6 (6)	H21A—C21—H21C	110.00
C6—C4—C7	109.0 (7)	H21A—C21—H21B	109.00
O1—C4—C5	111.1 (6)	C22—C21—H21C	109.00
C5—C4—C7	112.5 (6)	C21—C22—H22A	112.00
O3—C8—N1	123.4 (6)	C20—C22—H22B	105.00
N1—C8—C9	115.6 (6)	C23A—C22—H22B	124.00
O3—C8—C9	120.9 (6)	C21—C22—H22B	105.00
C8—C9—C20	111.6 (5)	C23A—C22—H22A	112.00
N2—C9—C8	112.3 (5)	H22A—C22—H22B	13.00
N2—C9—C20	112.5 (5)	C20—C22—H22A	112.00
N2—C14—C15	119.6 (6)	C23B—C22—H22A	86.00
O4—C14—C15	115.0 (6)	C23B—C22—H22B	99.00
O4—C14—N2	125.3 (6)	C22—C23A—H23A	110.00
N3B—C15—C14	107.6 (17)	C22—C23A—H23B	109.00
C14—C15—C16	110.3 (6)	C22—C23A—H23C	110.00
N3A—C15—C14	106.7 (14)	C22—C23A—H23D	147.00
N3A—C15—C16	100.8 (12)	C23B—C23A—H23C	77.00
N3B—C15—C16	115.1 (15)	C23B—C23A—H23D	119.00
C15—C16—C18	110.3 (6)	C23B—C23A—H23F	44.00
C17—C16—C18	109.3 (6)	H23A—C23A—H23B	109.00
C15—C16—C17	111.7 (6)	H23A—C23A—H23C	109.00
F6—C17—C16	112.3 (6)	H23A—C23A—H23D	101.00
F5—C17—F6	104.3 (6)	H23A—C23A—H23F	33.00
F4—C17—C16	114.6 (6)	H23B—C23A—H23C	109.00
F4—C17—F5	107.0 (6)	H23B—C23A—H23D	70.00
F4—C17—F6	107.5 (6)	H23B—C23A—H23F	138.00
F5—C17—C16	110.5 (6)	H23C—C23A—H23D	47.00
F1—C18—F3	105.4 (7)	H23C—C23A—H23F	104.00
F2—C18—F3	105.6 (6)	C22—C23A—H23F	82.00
F3—C18—C16	112.3 (5)	C23B—C23A—H23A	75.00
F2—C18—C16	112.3 (7)	C23B—C23A—H23B	169.00
F1—C18—F2	107.5 (5)	H23D—C23A—H23F	121.00
F1—C18—C16	113.2 (6)	C22—C23B—H23E	110.00
C9—C20—C22	114.7 (6)	C22—C23B—H23F	109.00
C20—C22—C23A	104.1 (8)	C22—C23B—H23D	109.00
C23A—C22—C23B	26.5 (10)	C23A—C23B—H23C	62.00
C20—C22—C21	109.8 (7)	C23A—C23B—H23D	20.00
C21—C22—C23B	111.8 (10)	C23A—C23B—H23A	63.00
C21—C22—C23A	107.5 (9)	C23A—C23B—H23F	105.00
C20—C22—C23B	123.2 (10)	H23A—C23B—H23C	97.00
C22—C23A—C23B	60.0 (17)	H23A—C23B—H23D	66.00
C22—C23B—C23A	94 (2)	H23A—C23B—H23E	128.00
O5A—C24A—C25A	106.5 (16)	H23A—C23B—H23F	43.00
O5B—C24B—C25B	114.9 (19)	H23C—C23B—H23D	43.00
O6A—C25A—C24A	109.9 (15)	C23A—C23B—H23E	128.00
O6B—C25B—C24B	116 (2)	H23C—C23B—H23F	129.00
N1—C2—H2	109.00	H23D—C23B—H23E	109.00
C1—C2—H2	109.00	H23D—C23B—H23F	109.00
C3—C2—H2	109.00	H23E—C23B—H23F	109.00
C2—C3—H3A	109.00	H23C—C23B—H23E	66.00
H3A—C3—H3C	109.00	C22—C23B—H23A	121.00
H3B—C3—H3C	109.00	C22—C23B—H23C	120.00
C2—C3—H3B	109.00	O5A—C24A—H24A	110.00
C2—C3—H3C	109.00	O5A—C24A—H24B	110.00
H3A—C3—H3B	109.00	C25A—C24A—H24A	110.00
C4—C5—H5A	109.00	C25A—C24A—H24B	110.00
C4—C5—H5B	110.00	H24A—C24A—H24B	109.00
C4—C5—H5C	109.00	O5B—C24B—H24C	109.00
H5B—C5—H5C	109.00	C25B—C24B—H24D	108.00
H5A—C5—H5B	110.00	O5B—C24B—H24D	109.00
H5A—C5—H5C	109.00	C25B—C24B—H24C	109.00
C4—C6—H6A	109.00	H24C—C24B—H24D	108.00
C4—C6—H6B	109.00	O6A—C25A—H25A	110.00
C4—C6—H6C	109.00	O6A—C25A—H25B	110.00
H6A—C6—H6B	110.00	C24A—C25A—H25B	110.00
H6B—C6—H6C	109.00	H25A—C25A—H25B	108.00
H6A—C6—H6C	110.00	C24A—C25A—H25A	110.00
C4—C7—H7C	109.00	O6B—C25B—H25C	108.00
H7A—C7—H7B	109.00	O6B—C25B—H25D	108.00
H7B—C7—H7C	109.00	C24B—C25B—H25C	108.00
C4—C7—H7A	110.00	C24B—C25B—H25D	109.00
H7A—C7—H7C	109.00	H25C—C25B—H25D	107.00
			
C4—O1—C1—O2	−4.5 (11)	C8—C9—C20—C22	165.1 (6)
C4—O1—C1—C2	170.9 (6)	O4—C14—C15—N3A	57.1 (13)
C1—O1—C4—C5	62.9 (8)	O4—C14—C15—C16	−51.5 (8)
C1—O1—C4—C6	−61.4 (8)	N2—C14—C15—N3A	−118.8 (12)
C1—O1—C4—C7	−176.9 (6)	N2—C14—C15—C16	132.6 (6)
C24A—O5A—N3A—C15	132.9 (18)	N3A—C15—C16—C17	55.9 (14)
N3A—O5A—C24A—C25A	−72 (2)	N3A—C15—C16—C18	177.7 (13)
C8—N1—C2—C1	−134.2 (6)	C14—C15—C16—C17	168.4 (6)
C8—N1—C2—C3	104.4 (7)	C14—C15—C16—C18	−69.9 (8)
C2—N1—C8—O3	2.2 (10)	C15—C16—C17—F4	41.6 (9)
C2—N1—C8—C9	178.7 (6)	C15—C16—C17—F5	−79.4 (8)
C14—N2—C9—C8	−105.0 (6)	C15—C16—C17—F6	164.6 (6)
C14—N2—C9—C20	128.2 (6)	C18—C16—C17—F4	−80.8 (8)
C19—N2—C9—C8	74.6 (7)	C18—C16—C17—F5	158.3 (7)
C19—N2—C9—C20	−52.3 (8)	C18—C16—C17—F6	42.3 (9)
C9—N2—C14—O4	−1.8 (9)	C15—C16—C18—F1	−76.2 (8)
C9—N2—C14—C15	173.6 (5)	C15—C16—C18—F2	161.9 (6)
C19—N2—C14—O4	178.6 (6)	C15—C16—C18—F3	43.0 (9)
C19—N2—C14—C15	−6.0 (9)	C17—C16—C18—F1	47.0 (9)
O5A—N3A—C15—C14	55.9 (18)	C17—C16—C18—F2	−75.0 (8)
O5A—N3A—C15—C16	171.0 (14)	C17—C16—C18—F3	166.2 (7)
O1—C1—C2—N1	169.0 (5)	C9—C20—C22—C21	177.4 (6)
O1—C1—C2—C3	−69.6 (7)	C9—C20—C22—C23A	−67.8 (9)
O2—C1—C2—N1	−15.5 (10)	C9—C20—C22—C23B	−47.6 (13)
O2—C1—C2—C3	105.8 (8)	C20—C22—C23A—C23B	140 (2)
O3—C8—C9—N2	−152.0 (6)	C21—C22—C23A—C23B	−104 (2)
O3—C8—C9—C20	−24.6 (8)	C20—C22—C23B—C23A	−49 (2)
N1—C8—C9—N2	31.5 (8)	C21—C22—C23B—C23A	86 (2)
N1—C8—C9—C20	158.8 (6)	O5A—C24A—C25A—O6A	−60 (2)
N2—C9—C20—C22	−67.7 (8)		

### Hydrogen-bond geometry (Å, °)

**Table:**

*D*—H···*A*	*D*—H	H···*A*	*D*···*A*	*D*—H···*A*
N3A—H3A1···F5	0.88	1.94	2.60 (3)	130
N1—H1N···O2	0.88 (5)	2.37 (7)	2.720 (9)	104 (5)
N1—H1N···O5A	0.88 (5)	2.38 (6)	3.245 (17)	169 (6)
